# A Universal Principle Illustrated

**Published:** 1919-10-18

**Authors:** 


### NURSES AND THE HEALTH MINISTRY.

### A Universal Principle Illustrated.

The letter addressed by the Secretary of the College of Nursing to the Minister of Health is opportune and interesting, and, as Miss Rundle observes, expresses the opinion of the whole profession.

That the nursing profession is not adequately represented is, to me at least, not a matter for surprise. I wonder if it has occurred to anybody to inquire into the causes for the omission? Labourers are represented in the British Cabinet, and their voices are heard when vital questions concerning the Empire are being decided. In forming Consultative Councils the Ministry of Health would appear to have forgotten the nurses.

I am not in a position to state positively the cause for this apparent lapse of memory, but I think I can offer a very probable and reasonable explanation. I read a good deal of literature emanating from the Ministry within recent months, and in this it cannot be said that the nurse has been overlooked or forgotten. On the contrary, it is made perfectly plain that without her services the work of the Department will be seriously handicapped. But the nurses, it is explained, must be specially trained, and, if this is not done, others than nurses will have to be employed. I set all this out in a series of articles in The Hospital, and endeavoured to demonstrate that the opportunity is a golden one for the profession. The position of health visitor will be well paid, and the work of a most useful and interesting character. So strongly emphasised is all this in the Ministry's pamphlets that the author might have been a nurse writing in the interests of her class.

May I inquire what have the nurses' leaders been doing about all this? Have they endeavoured in any way to bring the machinery of nurse training-schools into alignment with the national demand, or are they leaving the harvest that ought to be reaped by their own people to outsiders trained in women's colleges? One would have thought that immediately this attractive bill of fare was made out by a great public department the leaders of the nurses would have become extremely busy, and that deputations would have been daily in waiting on Hospital Committees with plans and arrangements showing how the present system might be adjusted to suit the new requirements. If nothing has been attempted in this direction, I venture to suggest that the Ministry may have arrived at the natural conclusion either that those ladies who think their claims have been overlooked lack interest in the concerns of the rank and file, or else lack that administrative and constructive ability which would render their services indispensable.

Take another case. The Minister of Health recently referred to the scandalous manner in which nurses are underpaid for their services to the nation. This is not a matter of to-day or yesterday. The cry has been going up for years, and in many cases a certain amount of relief has come. In all these years no organised or unified effort has ever been made by any school of nurses' leaders to impose their will on the employers of nurses, or even to represent to them the claims of the workers for better conditions of service. The relief that has come is the result of natural causes, often local causes. The nurses themselves have either submitted their claims "on their own," or else Hospital Committees found it impossible to recruit their staffs on the old scale.

Not by marked inactivity in vital matters have the British labourers succeeded in ensuring for their representative a place of honour in the Cabinet. To the credit of the Labour leaders stands a long catalogue of Acts of Parliament protecting the workers and ensuring their continually improving conditions of employment. It is a long story and goes back more than half a century, and during all this time the number of Labour representatives in the Imperial Parliament has been increasing. They have not arrived there on invitation from the Sovereign or the Prime Minister. They are there simply because they have demonstrated that they cannot be done without. They are a power and a force in the land, and no great enterprise can be undertaken without their counsel and co-operation.

I mention this merely as an illustration of a universal principle. And I venture to suggest that it is just possible that the authorities in the Department of the Ministry of Health may not have received convincing evidence that such an addition to their Councils as Miss Rundle requests is essential. This application may and ought to be granted. But if it is, let there be no doubt that if such representation is to be of real value to the profession there will have to be greater activity on behalf of the rank and file. In other words, if British nurses are not organised and trained in sufficient numbers to satisfy the requirements of the Ministry of Health, their representatives can fulfil no useful function on Consultative Boards.

IERNE.

